# Cut-off scores for sensitivity interpretation of the Korean Highly Sensitive Person Scale

**DOI:** 10.1371/journal.pone.0309904

**Published:** 2024-09-19

**Authors:** Wonyoung Yang, Miji Kwon

**Affiliations:** 1 Division of Architecture, Gwangju University, Gwangju, Korea; 2 Department of Speech-Language Pathology, Gwangju University, Gwangju, Korea; University Magna Graecia of Catanzaro, ITALY

## Abstract

This study aimed to replicate findings on sensitivity groups, their proportions, and cut-off scores in a South Korean population. Uniquely, it extended the age range to include participants up to 80 years old, representing the first attempt to validate these constructs across such a broad age spectrum in this cultural context. A total of 1773 South Koreans in their 20s to 80s participated in the Highly Sensitive Person Scale (HSPS) questionnaire survey, conducted to establish a cut-off score to be used more conveniently in real-world scenarios. The results showed that 22.0%, 45.3%, and 32.7% belonged to the low-, medium-, and high-sensitivity groups, respectively. The average item scores of 3.81 and 4.73 served as cut-off points distinguishing low- from medium-sensitivity and medium- from high-sensitivity groups, respectively. This study represents applied research on the use of HSPS. Research on HSPS cut-off scores considering cultural or demographic characteristics is still in its early stages, and accumulating data through various surveys is key for in-depth comparative analyses.

## Introduction

Sensory processing sensitivity (SPS) is a personality trait that describes concurrent heightened awareness and greater cognitive processing during sensory simulation [1]. SPS has been merged into a larger theoretical meta-framework known as environmental sensitivity [2, 3]. The central claim of environmental sensitivity theory is that humans can be classified according to the extent to which they register and process environmental stimulation; that is, their neurosensitivity [2, 4]. Environmental sensitivity is also defined as one of the most basic individual characteristics and may be observed not only in humans but also in most animal species [5]. It allows individuals to perceive, evaluate, and react to various physical and psychosocial environmental conditions. SPS has received considerable attention as a marker of environmental sensitivity [3].

SPS, operationalized through the Highly Sensitive Person Scale (HSPS), is the most direct attempt to measure neurosensitivity levels in humans [5]. It has been traditionally treated as a qualitative and dichotomous trait, based on which people have been categorized into two groups: approximately 20% with high SPS and the remaining 80% [1, 4]. However, prior studies [6–11] (Table 1) have reported that SPS is a quantitative and continuous trait with a normal distribution. They identified three sensitivity groups instead of two using a data-driven approach—latent class analysis (LCA)—and established preliminary cut-off scores for each group. Two early studies in the UK [5, 6] tested a group of children, adolescents, and university students. The university students and adolescents showed similar trends in the percentage of low- (30.5% and 35.0%, respectively), medium- (40.3% and 41.0%, respectively) and high-sensitivity (29.2% and 24.1%, respectively) mean scores (3.14, 4.14, and 5.02 vs. 3.00, 4.22, 5.06, respectively) of each group and cut-off scores (3.71 and 4.66 vs. 3.64 and 4.65, respectively; Table 1). However, in children aged 11 to 14 years, the percentage, mean, and cut-off scores for high-sensitivity group were higher: 34.1%, 5.39, and 4.15/4.75, respectively. Further, in this group, the percentage of those with low sensitivity was lower (24.7%). However, another children’s sensitivity study [9] reported different results, with approximately 42% of Polish children classified into the low-sensitivity group. Tillmann et al. [8] also reported a lower percentage of low-sensitivity group (14.3%) in their study of adolescents; however, less than 20% of low sensitivity was not often reported in other studies. These findings may provide useful information for psychological interventions that consider the individual differences in SPS. However, it remains unclear whether these findings can be generalized to samples from different social, cultural, and age groups.

**Table 1:** Cut-off scores for the three sensitivity groups based on HSPS.

No.	Study	Participant		HSPS instrument	Latent class analysis					Three latent classes % Mean score (SD)			Cut-off score	
		Language/ethnicity	N/age		Class	AIC	BIC	LMR-A	Entropy	Low	Medium	High	Low-medium	Medium-high
1	Lionetti et al. (2018) [6]	The USA and the UK/mixed ethnicity	906/university students	3-factor-27 7-point scale	3	88009.9	88538.29	< .01	.860	30.5% 3.14 (.45)	40.3% 4.14 (.33)	29.2% 5.02 (.43)	3.71	4.66
2	Pluess et al. (2018) [5]	The UK/mixed ethnicity	344/11–14 years 258/11–12 years	3-factor-12 7-point scale	3	24682.6	24896.35	< .001	.850	24.7% 3.68 (.80)	41.2% 4.24 (.67)	34.1% 5.39 (.63)	4.17	4.75
			1470/15–19 years	3-factor-12 7-point scale	3	63703.9	63968.51	< .001	.800	35.0% 3.00 (.51)	41.0% 4.22 (.45)	24.0% 5.06 (.63)	3.64	4.65
3	Son and Kim (2021) [7]	South Korean	477/university students	4-factor-18 7-point scale	3	9875.5	9986.48	.001	.714	19.5% -	54.7% -	25.7% -	Unclear	Unclear
				3-factor-10 7-point scale	3	-	-	-	-	20.3% -	52.7% -	27.0% -	Unclear	Unclear
4	Tillmann et al. (2021) [8]	German	749/grade 7–13	G-HSPS-10 4-point scale	3	-	18165.51	< .05	.760	14.3% 1.97 (.34)	53.3% 2.58 (.26)	32.4% 3.12 (.32)	-	-
5	Baryła-Matejczuk et al. (2022) [9]	Polish	928/10–14 years	P-HSPS-12 7-point scale	3	-	38035.26	-	.080	41.8% 3.98 (.56)	21.0% 4.87 (1.04)	37.7% 5.26 (.47)	-	-
6	May et al. (2022) [10]	South Africa/mixed ethnicity	750/university students	5-factor-20	3	69597.2	71851.83	-	.904	24.1% -	39.1% -	36.8% -	4.03	4.82
			1400/Birth to 20+ cohort	5-factor-20 7-point scale	3	68432.3	70991.43	-	.866	28.4% -	35.6% -	36.0% -	3.49	4.17
7	Yano and Oishi (2023) [11]	Japan	1257/university students	J-HSPS-19 7-point scale	3	79594.9	81361.82	< .001	.900	23.2% 3.29 (.55)	48.8% 4.13 (.40)	28.0% 5.04 (.48)	3.68	4.57
8	Yang and Kwon (the present study)	South Korean	1773/20s-80s	3-factor-27 7-point scale	3	161272.3	163947.0	0.0251	.913	22.0% 3.22 (.49)	45.3% 4.27 (.28)	32.7% 5.32 (.44)	3.81	4.73

Notes. AIC: Akaike information criterion; BIC: Bayesian information criterion; LMR-A: Lo–Mendell–Rubin-adjusted likelihood ratio; HSPS: Highly Sensitive Person Scale.

Tillman et al. [8] reported cultural differences in cut-off scores and percentage of each sensitivity group, while replicating the existence of the three sensitivity groups. Moreover, Japanese studies support cultural differences in SPS. Iimura and Kibe [12] found higher scores on the Highly Sensitive Child Scale in Japanese adolescents than in British adolescents and inferred that biological factors influenced this difference. Yano et al. [13] reported that Japanese adults had higher HSPS scores than German adults did. In line with the results of the Japanese studies, Son and Kim [7] identified three sensitivity groups; that is, high (approximately 26%), medium (approximately 55%), and low (approximately 19%), in South Korean university students. Their findings suggest that the percentage of South Korean university students with low sensitivity (19.5%) is lower than that of British university students (30.5%) [6], although the percentages of South Korean (25.7%) and British (29.2%) students with high sensitivity were similar. Unfortunately, the mean and cut-off scores for each sensitivity group could not be directly compared owing to unidentified data [7].

Additionally, most studies reported three groups with cut-off scores for young adults or adolescents (Table 1). Pluess et al. [5] and Baryła-Matejczuk et al. [9] studied children aged 10–14 years, Pluess et al. [5] and Tillmann et al. [8] studied adolescents, and university students were the most common age group in different HSPS studies [6, 7, 10, 11]. However, no studies looked at an age group older than university students in their early 20s.

The current study investigated whether the findings from other cultures and age groups regarding SPS can be replicated in South Korean samples. The research hypotheses are as follows:

1. There exist three distinct sensitivity groups (low, medium, and high) in the South Korean population, similar to those found in other cultures.
2. The percentages of individuals in each sensitivity group and the cut-off scores between groups in the South Korean sample will be comparable to those found in other studies, particularly for age groups older than university students in their early 20s.
3. There may be cultural differences in the distribution of sensitivity groups and cut-off scores between South Korean samples and those from other countries, particularly Western countries.

This study aims to establish cut-off scores that can be used more conveniently in real-world situations, focusing on a broader age range than previous studies, which have primarily concentrated on children, adolescents, and young adults.

## Methods

### Participants and procedures

A total of 1773 South Koreans in their 20s to 80s completed the HSPS questionnaire. As there were no missing items, the final sample comprised 1773 participants (929 women and 844 men) in the following age groups: 20s, 19.0%; 30s, 14.9%; 40s, 24.6%; 50s, 24.5%; and 60s, 16.9%. The sample was randomly divided into subgroups A (Sub A) and B (Sub B) of 886 and 887 participants, respectively, to permit a cross-validation approach to test and re-test and ensure whether the internal structure of the HSPS could be considered unidimensional and to validate the cut-off scores (Table 2).

**Table 2:** Descriptive statistics of the sample.

	Total		20s–30s		40s–50s		60s–80s	
	n	(%)	n	%	n	%	n	%
Total	1773	(100.0)	602	(34.0)	871	(49.1)	300	(16.9)
Men	844	(47.6)	237	(13.4)	417	(23.5)	190	(10.7)
Women	929	(52.4)	365	(20.6)	454	(25.6)	110	(6.2)
Subgroup A	886	(100.0)	300	(33.9)	436	(49.2)	150	(16.9)
Men	400	(45.1)	109	(12.3)	195	(22.0)	96	(10.8)
Women	486	(54.6)	191	(21.6)	241	(27.2)	54	(6.1)
Subgroup B	887	(100.0)	302	(34.0)	435	(49.0)	150	(16.9)
Men	444	(50.1)	128	(14.4)	222	(25.0)	94	(10.6)
Women	443	(49.9)	174	(19.6)	213	(24.0)	56	(6.3)

This study was conducted in December 2023. Participation was voluntary and anonymous. All participants provided informed consent before responding to the questionnaire. They were informed that they could withdraw from this study at any time without providing any justification. This study was conducted in accordance with the guidelines of the Declaration of Helsinki and approved by the Institutional Review Board of Gwangju University. The questionnaire was created using Google Forms and distributed through social media in South Korea. Participants completed the questionnaires, which accompanied instructions, on their own mobile devices or computers, and were compensated for their participation. Paper and online responses were assumed to be equivalent [14, 15].

### Measure: Highly Sensitive Person Scale

The Korean version of the HSPS [16], an adaptation of the HSPS [1] developed by Aron and Aron, was used in this study. The HSPS contains 27 statements, rated on a seven-point Likert scale ranging from 1 (*not at all*) to 7 (*extremely*).

### Data analysis

Statistical analyses were performed using Minitab 21.4.2 (Minitab LLC., State College, PA, USA), SPSS 29.0.2.0 (IBM Inc., Amrok, NY, USA)., and Mplus 8.10 (Muthen & Muthen, Los Angeles, CA, USA) [17].

#### Confirmatory factor analysis

Given the large sample size, a confirmatory factor analysis (CFA) was conducted with Sub-A (discovery sample) and Sub-B (replication sample) and another on the total sample based on the three component structure comprising ease of excitation (EOE), aesthetic sensitivity (AES), and low sensory threshold (LST) [18].

The four models (Table 3) were subjected to a CFA. The parameters were estimated using a robust maximum likelihood estimation method. Model comparison was guided by the following criteria [6]: (a) a qualitative evaluation of the fit indices of each model; (b) the comparative fit index (CFI) criterion—according to which, if the difference in the CFIs between two nested models is smaller than |0.01|, the hypothesis of no difference in fit between the two competing models should not be rejected; and (c) scaled chi-square difference tests. For the evaluation of the model fit indices, two relative indices—the Tucker–Lewis index (TLI) and CFI—and two absolute fit indices—the root mean square error of approximation (RMSEA) and standardized room mean square residuals (SRMR)—were computed. CFI and TLI values of > 0.95 and > 0.97, respectively, indicated acceptable and good fit, respectively. In addition, RMSEA from 0.05 to 0.08 reflected an adequate fit and SRMR < 0.08 reflected a good fit [19].

**Table 3:** Models for CFA.

Model	Description	Factors	Item no.
1	1-factor, Aron and Aron [1]	F1 (27)	1–27
2	3-factor, Smolewska et al. [18]	F1 (12): EOE	3, 4, 13, 14, 16, 17, 20, 21, 23, 24, 26, 27
		F2 (7): AES	2, 5, 8, 10, 12, 15, 22,
		F3 (6): LST	6, 7, 9, 18, 19, 25
3	3-factor, all 27 items	F1 (12): EOE	3, 4, 13, 14, 16, 17, 20, 21, 23, 24, 26, 27
		F2 (7): AES	2, 5, 8, 10, 12, 15, 22,
		F3 (8): LST	1, 6, 7, 9, 11, 18, 19, 25
4	4-factor, Son and Kim [7]	F1 (5): EOE	14, 16, 19, 25, 26
		F2 (4): AES	8, 10, 15, 22
		F3 (6): LST	1, 4, 7, 9, 13, 25
		F4 (3): Tact	2, 3, 17

Notes. CFA: confirmatory factor analysis.

#### Latent class analysis and cut-off scores

To test for the distinct sensitivity categories, LCAs were conducted on all HSPS items, models with 1–5 classes in Sub A and Sub B, as well as in the total sample. The optimal number of classes was determined based on the following criteria [6]: a) Akaike information criterion (AIC), (b) Bayesian information criterion (BIC), (c) Lo-Mendell-Rubin-adjusted likelihood ratio (LMR-A), and entropy. AIC and BIC are comparative indices, and the lower the values, the better the model. LMR-A compares the fit of a specified class solution to a model with one or fewer classes. A significant p-value suggests that the specified model provides a better fit to the data than a more parsimonious model. Entropy refers to the confidence with which individuals can be categorized into different classes, with values approaching one indicating a clear delineation of membership [20].

After identifying the optimal number of classes, we investigated the distribution and overlap between different sensitivity classes to determine the preliminary cut-off scores. Cut-off scores were identified based on the LCA results in Sub A and were applied to Sub B to test for specificity and sensitivity.

## Results

### Preliminary analysis

The data appeared normally distributed, with a bimodal pattern indicated by two emerging peaks with a small dip between them. A CFA was conducted based on the four models listed in Table 4. The 3-factor model with all 27 items yielded a good fit (χ^2^ = 3472.72, CFI = .800, TLI = .782, RMSEA = .074, SRMR = .066; Table 4). The HSPS showed good internal consistency, with Cronbach’s alpha = 0.904 (Sub-A: 0.908; Sub-B: 0.901) for the 27 items in this study.

**Table 4:** CFA results.

	Description	χ^2^	CFI	TLI	RMSEA	SRMR
Total	1-factor, all 27 items	(324) 4662.59	0.725	0.702	0.087	0.069
	3-factor, Smolewska et al.	(323) 4921.78	0.709	0.683	0.090	0.129
	**3-factor, all 27 items**	**(321) 3472.72**	**0.800**	**0.782**	**0.074**	**0.066**
	4-factor, Son and Kim	(327) 6670.55	0.598	0.569	0.105	0.183
Subgroup A	1-factor, all 27 items	(324) 2628.96	0.720	0.696	0.090	0.073
	3-factor, Smolewska et al.	(323) 2665.79	0.715	0.691	0.090	0.131
	**3-factor, all 27 items**	**(321) 1977.96**	**0.799**	**0.780**	**0.076**	**0.069**
	4-factor, Son and Kim	(327) 3792.25	0.579	0.548	0.109	0.191
Subgroup B	1-factor, all 27 items	(324) 2515.40	0.717	0.693	0.087	0.070
	3-factor, Smolewska et al.	(323) 2745.80	0.687	0.660	0.092	0.130
	**3-factor, all 27 items**	**(321) 1968.40**	**0.787**	**0.767**	**0.076**	**0.068**
	4-factor, Son and Kim	(327) 3374.56	0.606	0.577	0.103	0.179

Notes. the best-fit model is in bold; CFA: confirmatory factor analysis; CFI: comparative fit index; TLI: Tucker–Lewis index; RMSEA: root mean square error of approximation; SRMR: standardized room mean square residuals.

### Latent class analysis

A LCA was performed using the measures of fit for 1- to 5-class solutions, as listed in Table 5. The LMR-A and entropy results showed that the 3-class solution fit the data better than the 2-class solution. Additionally, Sub B results were consistent with those of Sub A; therefore, we adopted the 3-class solution. In the 20s–30s and 40s–50s, the 3-class solutions fit better than any 2-class solution. However, in the 60s–80s, only two groups were observed.

**Table 5:** LCA results.

	Classes	AIC	BIC	SABIC	LMR-A	(p)	Entropy
Total							
	one	173197.8	174085.6	173571.0			
	two	165620.2	167401.4	166368.9	7896.4	(.017)	0.894
	**three**	**161272.3**	**1639478.0**	**162396.5**	**4670.1**	**(.025)**	**0.913**
	four	159079.5	162647.3	160579.1	2516.8	(.058)	0.911
	five	157703.5	162164.5	159578.5	1700.7	(.775)	0.922
Subgroup A							
	one	86522.8	87298.2	86783.8			
	two	82840.3	84396.0	83363.8	4004.9	(< .0005)	0.907
	**three**	**80621.0**	**82956.9**	**81407.1**	**2543.0**	**(.004)**	**0.928**
	four	79474.0	82590.1	80522.7	1471.7	(.234)	0.95
	five	92667.6	96698.3	94113.0	1166.6	(.764)	0.944
Subgroup B							
	one	86826.7	87602.4	87087.9			
	two	82920.9	84477.0	83444.8	4227.7	(< .0005)	0.918
	**three**	**81068.9**	**83405.4**	**81855.6**	**2176.1**	**(< .0005)**	**0.931**
	four	80088.7	83205.6	81138.2	1305.0	(.819)	0.94
	five	79510.6	83407.9	80822.8	910.2	(.761)	0.943
20s–30s							
	one	59341.6	60054.4	59540.1			
	two	57024.3	58454.3	57422.6	2640.8	(< .0005)	0.946
	**three**	**55576.5**	**57723.8**	**56174.6**	**1772.1**	**(.003)**	**0.951**
	four	54920.3	57784.8	55718.1	981.3	(.760)	0.962
	five	54650.5	58232.3	55648.1	599.7	(.769)	0.96
40s–50s							
	one	83864.2	84636.9	84122.5			
	two	79833.2	81383.4	80351.3	4352.8	(< .0005)	0.923
	**three**	**77986.2**	**80313.8**	**78764.0**	**2171.1**	**(< .0005)**	**0.93**
	four	76916.1	80021.1	77953.7	1400.8	(.770)	0.942
	five	76527.8	80410.3	77825.2	711.4	(.763)	0.938
60s–80s							
	one	29382.6	29982.6	29468.8			
	**two**	**28061.3**	**29265.0**	**28234.3**	**1645.6**	**(.008)**	**0.955**
	three	27385.4	29192.8	27645.2	999.1	(.222)	0.966
	four	27137.0	29548.2	27483.6	573.7	(.765)	0.975
	five	27093.3	30108.2	27526.7	391.9	(.781)	0.99

Notes: the best-fit solution is in bold; LCA: latent class analysis; AIC: Akaike information criterion; BIC: Bayesian information criterion; SABIC: sample-size-adjusted BIC; LMR-A: Lo-Mendell-Rubin-adjusted likelihood ratio.

### Means and cut-off scores

**Fig 1:**
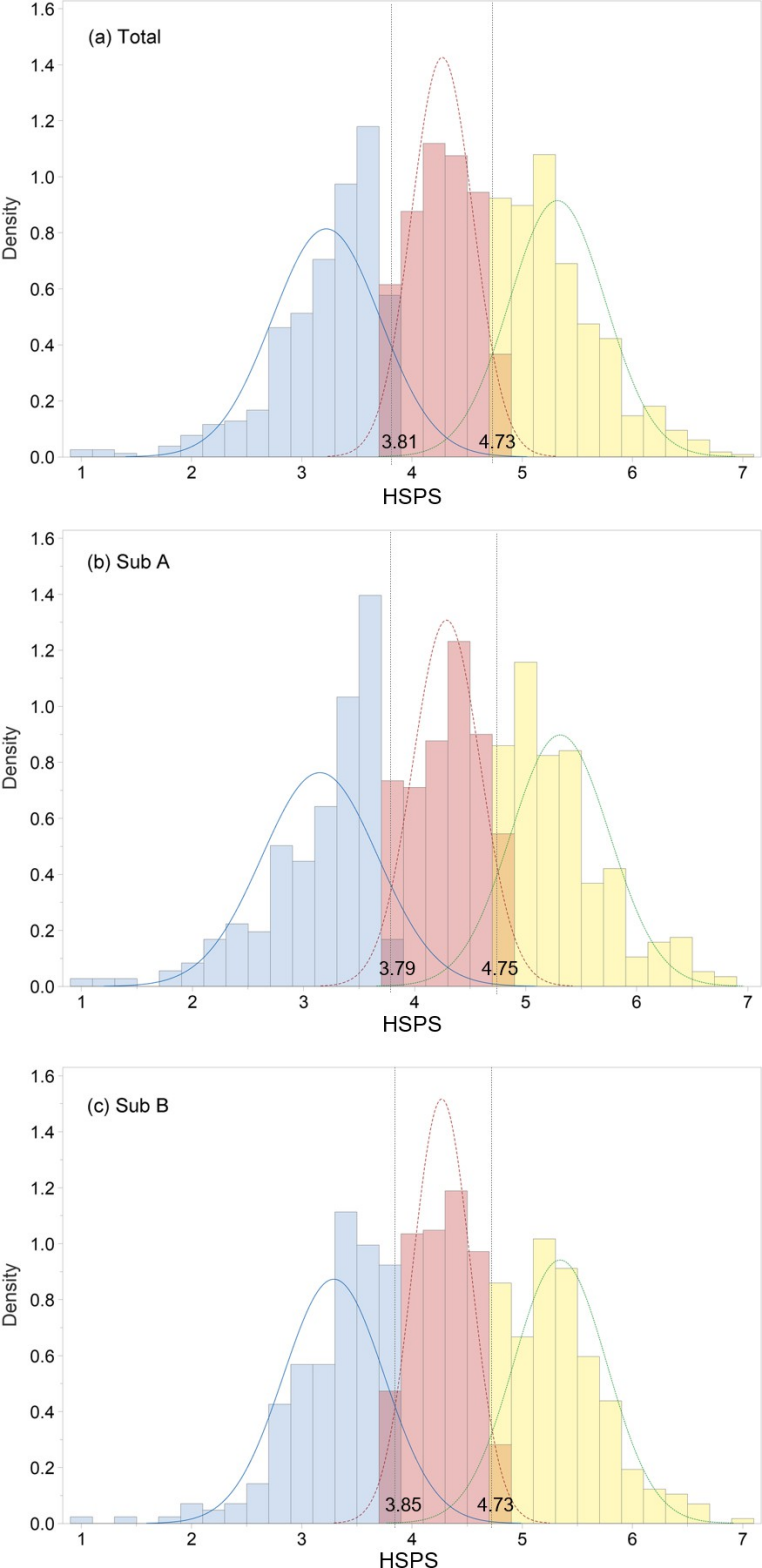
Distribution of HSPS mean scores in the three sensitivity groups. (a) Total, (b) Sub A, and (c) Sub B. HSPS: Highly Sensitive Person Scale; Sub A: subgroup A; Sub B: subgroup B.

**Fig 2:**
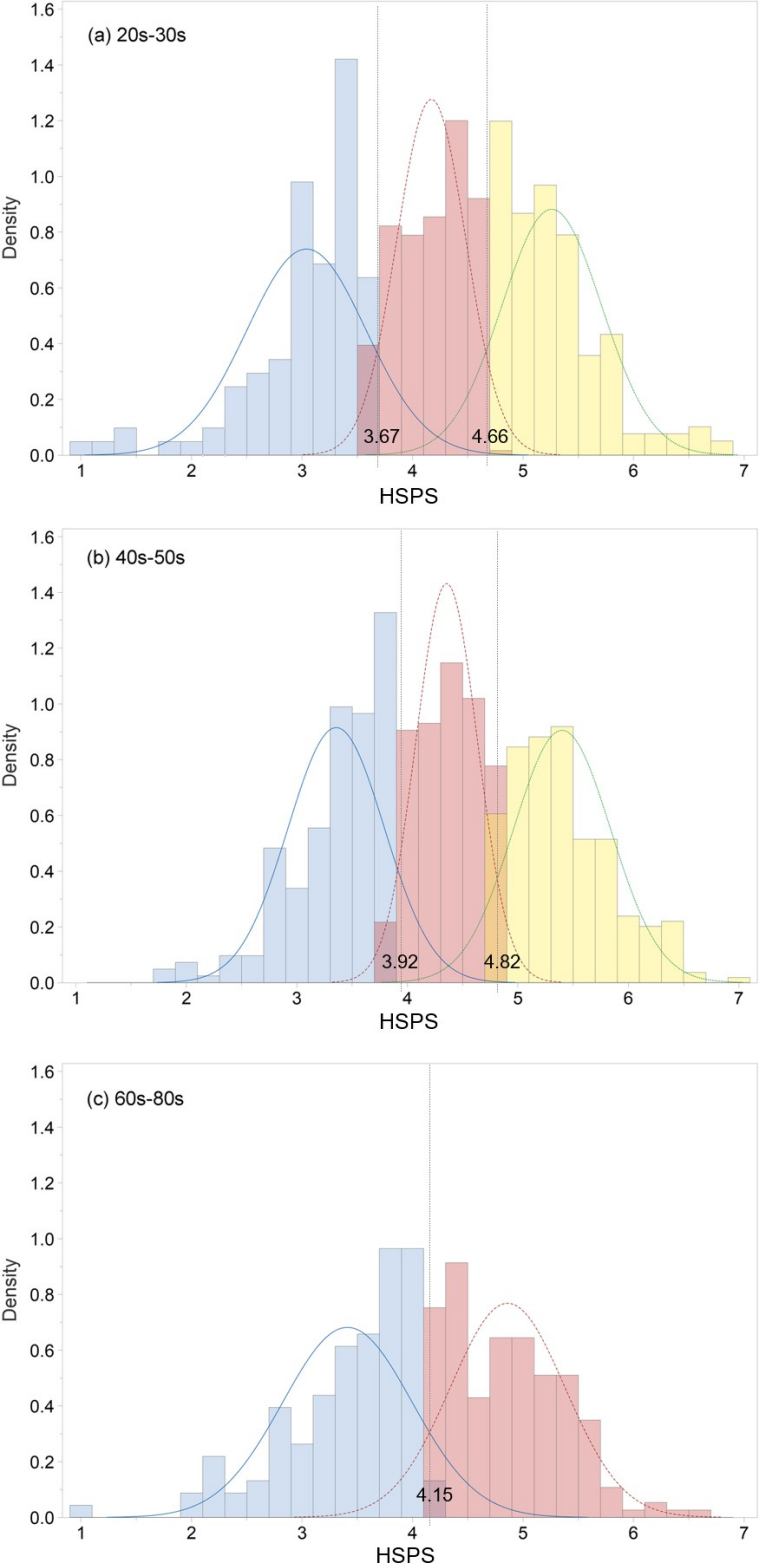
Distribution of HSPS mean scores by age group. (a) 20s-30s, (b) 40s-50s, and (c) 60s-80s. HSPS: Highly Sensitive Person Scale; Sub A: subgroup A; Sub B: subgroup B.

Based on the combined density plots (Figs 1 and 2), these three classes reflected low-, medium-, and high-sensitivity groups akin to “dandelion,” “tulip,” and “orchid” [6]. Accordingly, 22.0%, 45.3%, and 32.7% of participants were categorized as low-, medium-, and high-sensitivity groups, respectively. The average item scores of 3.81 and 4.73 served as cut-off points distinguishing low- from medium-sensitivity and medium- from high-sensitivity groups, respectively.

The cut-off scores of the three factors in the HSPS were also calculated. The mean and cut-off scores of those in their 40s–50s were greater than those in the 20s–30s and 60s–80s age groups. Among those in their 60s–80s, 38.0% were categorized into the low-sensitivity group and 62.0% were categorized into the high-sensitivity group, with a cut-off average score of 4.15. The means and cut-off scores for the EOE, AES, and low-sensitivity threshold were also calculated. The means of the EOE and AES of the low-, medium-, and high-sensitivity groups were similar to or slightly higher than the means of the overall HSPS, but the LST showed a relatively low average score distribution (Table 6).

**Table 6:** Means and cut-off scores of the sensitivity groups.

	Frequency	HSPS mean (SD)	3-factor mean (SD)			Cut-off score	
			EOE	AES	LST	Low-medium	Medium-high
Total		4.38 (0.86)	4.43 (0.96)	4.63 (0.94)	4.09 (1.12)	3.81	4.73
Low	22.00%	3.22 (0.49)	3.25 (0.68)	3.7 (0.86)	2.76 (0.70)		
Medium	45.35%	4.27 (0.28)	4.34 (0.51)	4.55 (0.68)	3.94 (0.60)		
High	32.66%	5.32 (0.44)	5.37 (0.53)	5.36 (0.67)	5.21 (0.71)		
Cut-off scores by factor			3.79, 4.85	4.01, 4.93	3.32, 4.55		
Subgroup A		4.39 (0.87)	4.43 (0.97)	4.65 (0.94)	4.10 (1.12)	3.79	4.75
Low	20.20%	3.15 (0.52)	3.16 (0.70)	3.66 (0.87)	2.68 (0.70)		
Medium	47.63%	4.29 (0.30)	4.34 (0.56)	4.59 (0.68)	3.96 (0.61)		
High	32.17%	5.31 (0.44)	5.35 (0.55)	5.36 (0.67)	5.21 (0.71)		
Subgroup B		4.38 (0.85)	4.45 (0.94)	4.61 (0.94)	4.08 (1.11)	3.85	4.73
Low	23.79%	3.29 (0.46)	3.35 (0.66)	3.73 (0.83)	2.82 (0.68)		
Medium	44.08%	4.27 (0.26)	4.35 (0.49)	4.52 (0.67)	3.93 (0.59)		
High	32.13%	5.34 (0.42)	5.39 (0.54)	5.39 (0.66)	5.23 (0.69)		
20s–30s		4.33 (0.86)	4.50 (0.96)	4.54 (0.95)	3.90 (1.11)	3.67	4.66
Low	16.94%	3.04 (0.54)	3.20 (0.77)	3.43 (0.76)	2.47 (0.69)		
Medium	50.50%	4.17 (0.31)	4.36 (0.54)	4.44 (0.72)	3.65 (0.58)		
High	32.56%	5.26 (0.45)	5.41 (0.58)	5.26 (0.69)	5.04 (0.75)		
40s–50s		4.44 (0.84)	4.46 (0.93)	4.71 (0.91)	4.19 (1.10)	3.92	4.82
Low	23.76%	3.36 (0.44)	3.37 (0.61)	3.87 (0.84)	2.89 (0.63)		
Medium	45.01%	4.36 (0.28)	4.38 (0.53)	4.63 (0.63)	4.08 (0.59)		
High	31.23%	5.40 (0.44)	5.41 (0.54)	5.46 (0.64)	5.33 (0.66)		
60s–80s		4.31 (0.89)	4.24 (0.98)	4.59 (1.00)	4.18 (1.13)	4.15	
Low	38.00%	3.41 (0.59)	3.33 (0.75)	3.85 (0.91)	3.14 (0.80)		
High	62.00%	4.86 (0.52)	4.79 (0.62)	5.04 (0.76)	4.82 (0.78)		

Notes. HSPS: Highly Sensitive Person Scale; EOE: ease of excitation; AES: aesthetics sensitivity; LST: low sensory threshold.

### Three-factor mean scores

**Fig 3:**
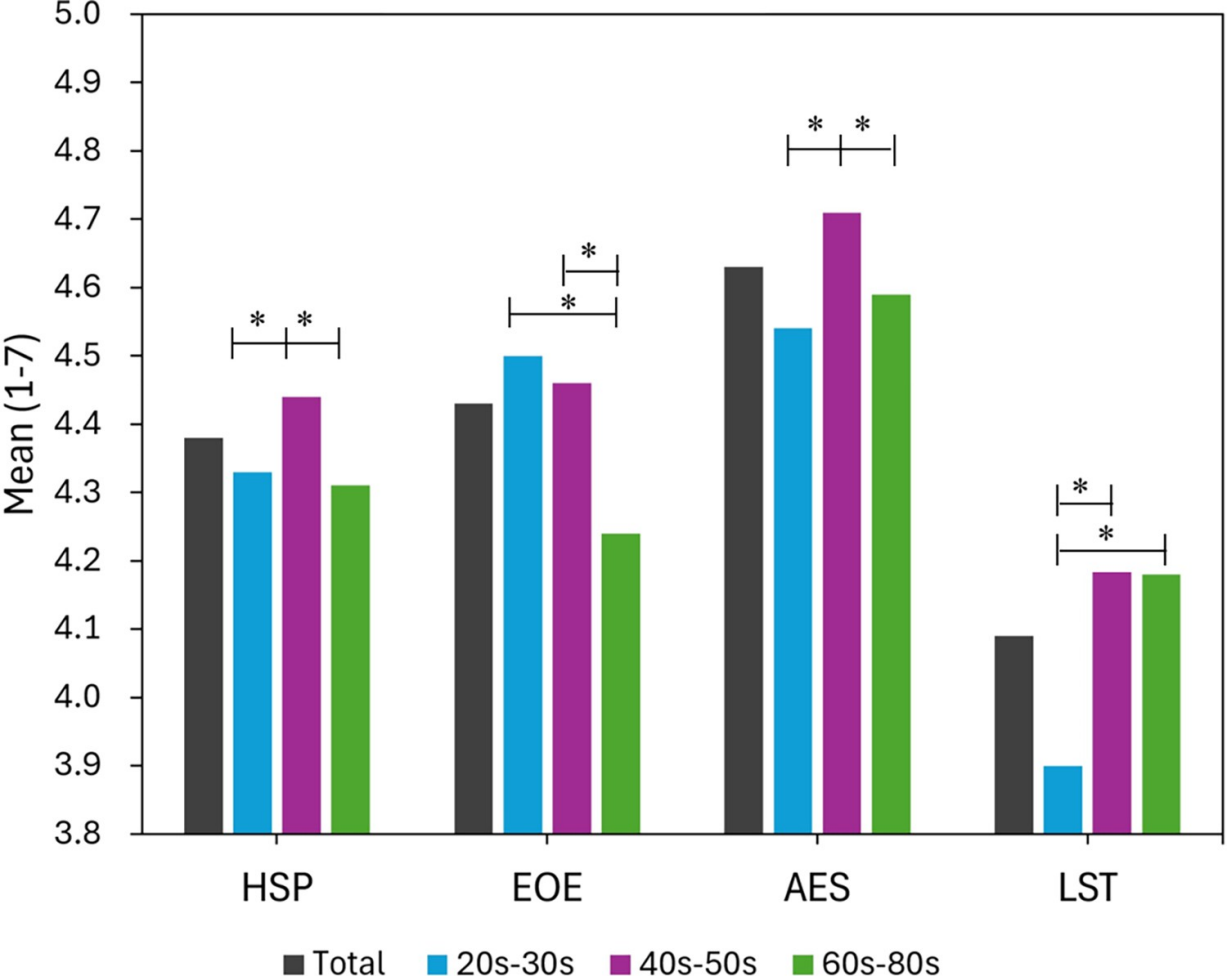
HSPS mean score and 3-factor scores by age (*p < .05). HSPS: Highly Sensitive Person Scale; EOE: ease of excitation; AES: aesthetic sensitivity; LST: low-sensitivity threshold.

In general, the mean score of the AES factor was higher than that of the EOE and LST factors (Fig 3). The LST ranked the lowest of the three. Especially in those in their 20s–30s, the LST score was significantly lower than that of the groups aged older than 40 years. However, the mean score of AES for those in their 20s–30s ranked the highest with statistical significance. In those in their 60s–80s, the mean score of EOE was significantly lower than that in other age groups.

## Discussion

### Cross-cultural comparison of sensitivity groups

The main contribution of this study is the identification of distinct sensitivity groups among South Koreans. The results replicated the three sensitivity groups, including the cut-off scores, which are consistent with the findings of prior HSPS studies (Table 1). The proportion of each sensitivity group differed slightly between the present and previous studies (Table 1).

In South Korean and Japanese studies, the proportion of sensitivity differed from that in British studies [6]. The proportion of each group in the Japanese sample [11] was similar to that in our sample. In addition, the proportion of each sensitivity group in Son and Kim’s [7] study was similar to that in our group in their 20s–30s. However, the proportions of high- and low-sensitivity groups were less and that of medium sensitivity group was more in their South Korean sample than those in our sample.

In Western studies, Lionetti et al.’s [6] and May et al.’s [10] adult samples belonged to high- and low-sensitivity groups more frequently and to medium sensitivity group less frequently than our sample. This is consistent with the findings of Yano and Oishi [11]. This trend can be explained by cultural differences in the responding to Likert-type scales [21]. Harzing [21] analyzed the data of university students from 26 countries and found that East Asians tend to select the middle option (i.e., “4” in a 7-point scale) more frequently than the extreme option (i.e., “1” or “7”). Research consistently showed that Western countries have a smaller percentage of moderately sensitive individuals compared to Eastern countries. This trend was also observed in Western studies of young people in Pluess et al. [6] and Baryła-Matejczuk et al. [9]. However, there are still insufficient results on HSPS cut-off scores to conclude that these differences between the East and the West are solely due to cultural differences in responding. Further, differences in these three factors between age groups were observed in this study. However, there are not enough research data to confirm this issue; therefore, additional research is needed.

### Comparative analysis of the three HSPS factors

**Fig 4:**
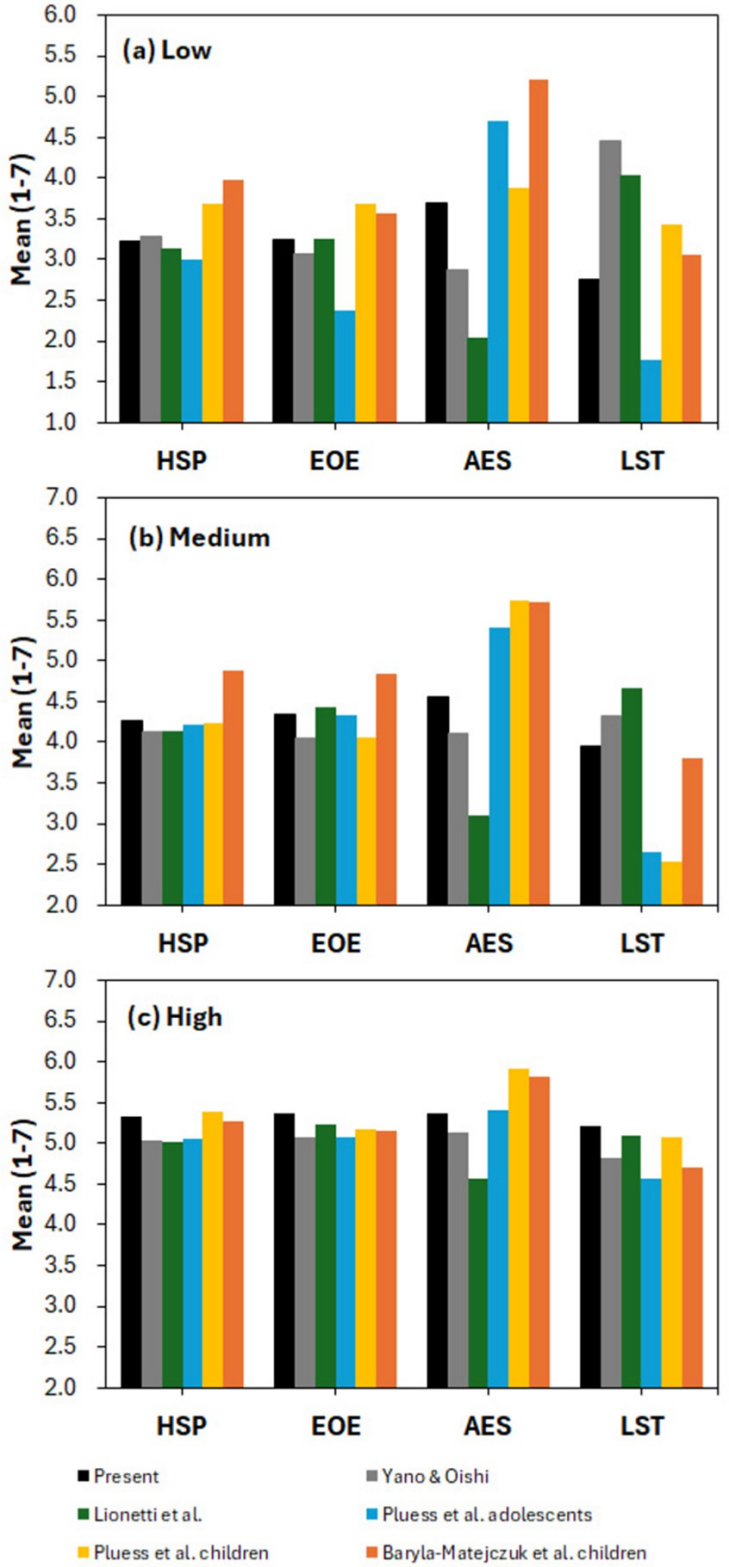
HSPS mean score and 3-factor scores of the previous studies. HSPS: Highly Sensitive Person Scale; EOE: ease of excitation; AES: aesthetic sensitivity; LST: low-sensitivity threshold.

HSPS scores across different populations, age groups, and sensitivity levels are compared in Fig 4. As expected, mean scores generally increase from low- to high-sensitivity groups across all measures, with the difference being most pronounced for the overall HSP score. In most studies, the mean AES score was the highest, with the exception of Lionetti et al. [6], in which the British participants showed the lowest mean AES score.

In the low-sensitivity group, AES scores are notably higher than other factors. The mean scores of children [5, 9] were higher than those of adults for all factors. The medium sensitivity group shows more consistency across factors, but AES still tends to be highest. Children and adolescents [5, 9] showed relatively low scores in the LST and high scores in AES. In the high-sensitivity group, scores are more uniform across factors and studies. Moreover, the mean scores for the three factors in the Japanese [11] individuals and South Korean participants in this study were similar, possibly indicating cultural aspects in sensitivity or reporting.

### Relationship between age and HSPS

This study investigated the relationship between age and HSPS. The findings revealed several key points. Age-related trends in HSPS scores showed that middle-aged adults (40–59 years) had the highest average scores, while younger (20–39 years) and older (≥ 60 years) adults exhibited relatively lower scores. The LCA indicated that a three-class model best fit the data for participants aged 20–59 years, whereas a two-class model was more suitable for those aged 60 years or older. Age differences were also observed in HSPS factors. Older adults (≥ 60 years) scored significantly lower in EOE. Young adults (20–39 years) scored significantly lower in LST. Middle-aged adults (40–59 years), the most sensitive group, scored significantly higher in AES. These results suggest that age plays a key role in SPS as measured by the HSPS.

To our knowledge, most studies on HSPS have focused on young people in their 20s, and very few studies have examined the relationship between age and HSPS.

Ueno et al. [22] reported linear relationships between age and SPS factors. They found that LST and EOE decreased with age, while AES increased. While our study also identified a relationship between age and SPS, we did not observe the linear trends reported by Ueno et al. This discrepancy in the findings warrants further investigation.

## Conclusions

A total of 1773 South Korean in their 20s to 80s participated in the HSPS questionnaire survey conducted to establish a cut-off score to be used more conveniently in real-world scenarios. The results showed that 22.0%, 45.3%, and 32.7% belonged to the low-, medium-, and high-sensitivity groups, respectively. The average item scores of 3.81 and 4.73 served as cut-off points distinguishing low- from medium-sensitivity and medium- from high-sensitivity groups, respectively. This study represents applied research on the use of HSPS. Research on HSPS cut-off scores considering cultural or demographic characteristics is still in its early stages, and accumulating data through various surveys is key for in-depth comparative analyses.
